# Hybrid apes in the Anthropocene: Burden or asset for conservation?

**DOI:** 10.1002/pan3.10214

**Published:** 2021-05-12

**Authors:** Alexandra Palmer, Volker Sommer, Josephine Nadezda Msindai

**Affiliations:** ^1^ School of Geography and the Environment University of Oxford Oxford UK; ^2^ UCL Anthropology University College London London UK

**Keywords:** conservation, genetics, great apes, hybrid, species, taxonomy

## Abstract

1. Conservationists often view hybrid animals as problematic, at least if anthropogenic influence caused the intermixing to occur. However, critics propose that humans should respect non‐human autonomy, reject and accept the creatures they have helped to create.
2. Based on two case studies of our own ethological, genetic and ethnographic research about chimpanzee and orangutan subspecies hybrids, we assess what, if anything, should be done about such animals. We consider problems posed by cross‐bred apes relating to: (a) *Breeding*—Do hybrids really experience reduced reproductive success? How are population‐level concerns and welfare of individual animals balanced in conservation breeding? (b) *Essentialism*—Are anti‐hybrid arguments based on essentialist or purist thinking? Does essentialism vary by conservation context? (c) *Pragmatism*—How do socio‐economic circumstances influence whether hybrids are embraced or ignored? Does the erosion of ‘untouched nature’ render hybrids more important?
3. We show that answers to these questions are complex and context‐specific, and that therefore decisions should be made on a case‐by‐case basis. For example, we find that anti‐hybrid arguments are essentialist in some cases (e.g. ape management in zoos) but not in others (e.g. ape reintroduction). Thus, rather than present recommendations, we conclude by posing nine questions that conservationists should ask themselves when making decisions about taxonomic hybrids.

​

A free Plain Language Summary can be found within the Supporting Information of this article.

## Abstract 2

A free Plain Language Summary can be found within the Supporting Information of this article.

## 1 INTRODUCTION

In conservation, hybrid animals are often viewed as problematic, at least if human influence led to the interbreeding (Allendorf et al., 2001). This reflects mainstream conservation philosophy, which seeks to preserve non‐human organisms and ecologies with minimal anthropogenic interference (Paquet & Darimont, 2010; Vucetich & Nelson, 2007). Conservationists have therefore aimed to prevent inter‐mixing between: domesticated and wild forms (e.g. domestic and Scottish wildcats, Fredriksen, 2016; domestic dogs and wolves; Peltola & Heikkilä, 2018); closely related animals encountering each other in human‐modified landscapes (e.g. coyote–wolf cross‐breeds or ‘coywolves’; Rutherford, 2018); and native populations with introduced animals (e.g. rainbow/westslope cutthroat trout hybrids in Western USA; Allendorf et al., 2001; released pet marmosets mixing with other *Callithrix* populations in Brazil; Malukiewicz, 2019).

As we move deeper into the Anthropocene, with humans increasingly shaping global climate and environments (Lewis & Maslin, 2018), critics of anti‐hybrid conservation policies propose that we should ‘love our monsters’ (to use Bruno Latour's phrase): care for the new creatures humans have generated (Rutherford, 2018), instead of culling or sterilising them (Fredriksen, 2016). Critics also consider anti‐hybrid policies as reflecting essentialist (purist) thinking (Biermann & Mansfield, 2014; Fredriksen, 2016; Rutherford, 2018), analogous to racism.

Related to such debates, several key questions emerge:

1. Does hybridisation affect breeding success?
2. Is anti‐hybrid management guided by essentialist thinking?
3. Should conservation in the Anthropocene aim to suppress taxonomic hybridisation?

​

We evaluate these concerns via two case studies of subspecies hybridisation in non‐human great apes (hereafter, apes), based on our own empirical research. While hybrids in general are ‘unloved’ conservation subjects (Mitchell, 2016), hybrid apes are less easily dismissed due to their liminal status as ‘almost human’ (Corbey, 2005; Cribb et al., 2014; Haraway, 1989). Because of this threshold position, the prospect of human‐ape cross‐breeding has been a subject of fascination and worry, with representations of human‐ape hybrids in art and literature serving as commentaries on human society, including ideas about race (Cribb et al., 2014; Sommer, 2007). Apes have also gathered particular attention in initiatives related to animal rights (Cavalieri & Singer, 1994) and species protection (Palmer, 2020). As a result, hybrid apes are best viewed as simultaneously loved and unloved conservation subjects.

A focus on apes is thus especially useful to guide thoughts about the aforementioned key concerns relating to hybrids: reproduction, essentialism and pragmatism. We do not ultimately aim for clear recommendations, but rather use our case studies to illustrate the complexity of the problem.

## 2 KEY PROBLEMS: BREEDING, ESSENTIALISM AND PRAGMATISM

Before engaging with our exemplary cases, we sketch out important facets of hybrid‐related debates.

### 2.1 Breeding

Lifetime reproductive output (an animal's number of offspring) is an indicator of biological *fitness*, which refers to the ability to survive and reproduce. Having offspring can also in some cases serve as a measure of animal *welfare*. Impaired reproduction might indicate poor health and welfare, in both wild and captive animals (Broom, 1991). Reproduction can also improve animal welfare by enabling the expression of parental—and in particular maternal—care. Thus, preventing reproduction (e.g. sterilisation, contraception) may have negative effects on animal welfare (Asa, 2016). Yet mating, gestation and lactation can also be stressful, and in captivity there is often an incentive to encourage rapid breeding without allowing females sufficient recovery time (Asa, 2016). Thus, in captive situations where humans control breeding, reproduction should not in itself be taken as a sign of good welfare (although it sometimes is, e.g. in farming contexts; Broom, 1991; Fraser, 2003).

In any case, hybridisation may have positive or negative effects on survival and reproduction. Those who view mixed breeds as negative will point to *outbreeding depression*, which is thought to compromise existing adaptations to local environments or to break up co‐adapted gene complexes, thereby creating ‘untested’, potentially harmful combinations. These effects may not manifest until the second generation, while first‐generation effects appear to be more common in plants than animals (Edmands, 2007). Intermixing incompatibility is generally more common in mammals and frogs than other taxa (Edmands, 2007). There is indeed empirical evidence for inter‐population variation causing chromosomal incompatibilities and thus sterility in otherwise healthy hybrids (e.g. spider monkeys, Ralls et al., 2013; owl monkeys, De Boer, 1982), or to the loss of local adaptations (e.g. hybrid ibex females birthed their calves too early in the season, leading to mass deaths; Frankham, 2010).

On the other hand, a lack of admixture can cause *inbreeding depression*, including expression of deleterious recessive traits (Keller & Waller, 2002; Lynch & Walsh, 1998) and reduced advantageous heterozygosity. These effects may manifest themselves in higher incidences of disease and parasite load (Frankham, 2010) or lower breeding success (e.g. in endangered New Zealand birds like the kakapo; White et al., 2015). Hybridisation is one way to mitigate inbreeding depression, when genes from other populations instil *hybrid vigour* or *heterosis* (Edmands, 2007). This upshot is capitalised upon in industries of livestock (e.g. ‘black baldy’ cattle; ‘blue butt’ pigs; ‘Cornish‐Rock’ poultry) and domestic pedigree dogs (Yordy et al., 2020), and in conservation, for example to aid the recovery of the highly inbred and isolated population of Florida panther (Whiteley et al., 2015). In addition, admixture of previously distinct lineages may be favoured by natural selection (Ackermann et al., 2019), revealing hybridisation as a ‘creative’ force for evolution (Biermann & Mansfield, 2014; Fredriksen, 2016). In transgressive segregation, a fresh hybrid lineage may colonise a novel niche (e.g. cross‐breeding of the now‐extinct Aurochs and Caucasian wisent produced the European bison; Węcek et al., 2017). Such *reticulate evolution* (Arnold & Meyer, 2006) is also evidenced by cross‐breeding between primate subspecies, species and even genera (e.g. *Eulemur* lemurs; Arnold & Meyer, 2006; *Hylobates* gibbons, Matsudaira & Ishida, 2020; cf. also Zinner et al., 2011). In baboons (*Papio* spp.), some populations consist of >30% hybrid individuals (Ackermann et al., 2006), and cross‐breeding may even happen with distinct genera (*Rungwecebus*, *Theropithecus*; Walker et al., 2019; Zinner et al., 2009).

Consequently, hybridisation is not only quite common, but often advantageous for survival, breeding and speciation. Despite such evidence, there are those who point to negative sides of cross‐breeding, advocating that it should be prevented, or that hybrids be culled or sterilised. In zoos, such fate is commonly suffered by ‘surplus’ animals, that is, those not wanted for breeding or exhibition (Asa, 2016). Hybrids may be deemed surplus based on the idea that captive populations should resemble wild counterparts (Hvilsom et al., 2013) so that zoos can act as ‘arks’ to repopulate the wild (Braverman, 2012). For example, a German zoo killed tiger cubs after discovering that their sire was not pure Siberian tiger, but rather a subspecies hybrid (Hance, 2010). Given that reproduction may positively impact welfare, animals are sometimes allowed to breed, to then have their offspring culled (Asa, 2016; Braverman, 2012). Veterinarians and zoo managers often maintain that ‘death is not a welfare issue’, provided animals are killed humanely. However, dissenters point out that preventing animals from curtailing positive forthcoming life stages does indeed harm their wellbeing (Yeates, 2010). Animal liberation and rights approaches similarly object to culls, on the grounds that animals are entitled to life and to an autonomous existence (Gruen, 2011).

Deciding what to do about hybrids therefore involves weighing population‐level concerns against the interests of individual animals (Vucetich & Nelson, 2007), for example preventing genetically impure animals from experiencing mating and raising offspring for the sake of population genetics. Prioritisation of populations at the expense of individuals has been referred to as ‘violent care’ (van Dooren, 2014). It has also been described as a typical, albeit controversial, approach to conservation biopolitics: management aimed at making certain non‐humans live and letting others suffer or die (Srinivasan, 2014).

Important questions to be asked about breeding are therefore:

1. Do hybrids really experience reduced reproductive success?
2. How are population‐level concerns and welfare of individual animals balanced in conservation breeding?

​

### 2.2 Essentialism

Hybrids break down conventional boundaries between taxonomic categories (‘liger’, ‘tigon’) and between nature and culture if domestic and wild animals cross‐breed (‘beefalo’; see also Fredriksen, 2016; Peltola & Heikkilä, 2018; Rutherford, 2018). Imagined hybrids can also dissolve borders between humans and animals (centaur, sphinx). Because such fusions challenge our ambiguity tolerance (Bryson et al., 2020), hybrids are often viewed as simultaneously captivating and unsettling, even monstrous (Haraway, 1985; Messy Beast, n.d.; Ritvo, 1997; Rutherford, 2018).

Taxonomy as a branch of biological science is linked to the fundamentally cultural practice through which humans seek to understand the world (Kirksey, 2015; Ritvo, 1997; Rutherford, 2018). The ordering of organisms is therefore conducted within social and political contexts. In Western tradition, its origins can be traced back to Plato and his conviction that categories are ‘real’ entities that exist in a perfect realm as ideal forms and eternal essences, while the world that we perceive is populated by imperfect incarnations of these ideals (Kraut, 2017; Zachos, 2016).

Many pre‐Darwinian taxonomists and philosophers, inspired by Plato, tended to adhere to such ‘essentialist’ view in which each single species is immutable and defined by a necessary and sufficient property—its ‘essence’—that each individual member shares (Zachos, 2016, 18). Still, while other pre‐Darwinian thinkers acknowledged variation within species and the potential for them to change over time (Richards, 2010; Wilkins, 2009; Winsor, 2006; Zachos, 2016), essentialist positions did still flare up in post‐Darwinian discourses. Importantly, the biological species concept (BSC; Dobzhansky, 1937; Mayr, 1942) treats species as more real and discrete than Darwin (Mallet, 2010). The classic framing of the BSC maintains that species are reproductively isolated through pre‐breeding (e.g. different mating behaviour) or post‐mating separation (e.g. hybrid inviability). In this view, hybridisation is negative for both individuals and their source populations and often implied to be a product of scarcity of conspecific mates (Ottenburghs et al., 2016; Detwiler et al., 2005) or accident (e.g. climate change bringing previously geographically isolated species into contact; Shurtliff, 2013). Alternatively, concepts of ‘speciation genes’ are invoked as causes of hybrid sterility and inviability (Coyne & Orr, 2004). Some contemporary philosophers also champion a ‘new biological essentialism’, according to which certain relationships among organisms and environments are necessary and sufficient for membership in a species, and that these associations constitute intrinsic species essences (see Ereshefsky, 2017). Natural historians are often at least implicitly touched by such ideas. Instead, as passionate nature lovers, they collect, group and name specimens of organisms—a hands‐on tradition on which today's conservationists often still rely (Biermann & Mansfield, 2014). In any case, essentialist views are necessarily static as they cannot easily accommodate cross‐overs and tend to invoke more or less stringent ideas of ‘purity’.

However, it should be remembered that the BSC—which casts hybridisation in a largely negative light—is by no means the only species definition, but that competing approaches are based on factors such as niche differentiation, genotypic clustering and morphology (reviewed in Zachos, 2016). Opinions are mixed on how to resolve the diversity of species concepts. *Monists* aim for one ‘true’ definition, arguing that all approaches ultimately get at the same thing (e.g. ‘unified species concept’, de Queiroz, 2005). *Pluralists* argue that no single correct definition exists, with radical views maintaining that the term ‘species’ is not a real category in nature (Ereshefsky, 2017; Zachos, 2016) or that we ought to abolish the Linnaean system of taxonomy altogether (Ereshefsky, 2000). A key problem lies in the fact that species definitions will necessarily impose a discrete ordering system onto continuous life forms and processes (Zachos, 2016)—a philosophically impossible task. Taxonomy will therefore always remain messy and debatable, notwithstanding its value for conversations about the diversity of life, and how forms change and relate to one another (Kirksey, 2015). In any case, given their effect on our assessment and understanding of biodiversity management, we need to continue to carefully scrutinise species concepts (Freudenstein et al., 2017).

At the minimum, current distinctions between species and other taxonomic units are not consistent. This becomes relevant in a political context where scarcity is valued over abundance in the natural world. Highly endangered species are likely to receive most attention in terms of conservation, creating an incentive to *split* rather than *lump* taxa (Agapow et al., 2004; Braverman, 2015; Mitchell, 2016). For example, relatively few previously unknown types of non‐human primate were discovered in their natural habitats during the last 50 years. Yet, based on largely loosely defined criteria relating to genetic markers, vocal communication, morphology or behaviour, many already described primate taxa were split, resulting in an increase in primate species from 180 in 1967 to 480 in 2013 (Zinner & Roos, 2016). As a case in point, in 1992 Madagascar was described as harbouring two species of the mouse lemur *Microcebus*; two decades later, the number of species had grown to at least 20 (Yoder et al., 2016), and may yet increase beyond the currently recognised 24 species (Schüßler et al., 2020). All these new species can likely interbreed. Thus, humans sometimes create the very categorical prerequisites without which a ‘hybridisation’ would not occur in the first place.

Essentialist thinking still easily crops up in conservation practice as the default, never mind that such views have become untenable in light of evolutionary theory (Ereshefsky, 2017). Biermann and Mansfield (2014, 266) point out that conservationists often insist ‘on the relative purity of species and the need to defend that purity from threats’ such as combining different organismic varieties and introducing non‐native species, despite their overall aim of fostering and promoting natural diversity. The authors propose that this apparently contradictory effort to maintain ‘purity within diversity’ (p. 268) resembles ideas about race in humans, echoing the frequently made accusation that anti‐invasive species sentiment is xenophobic and racist (Simberloff, 2003; Switzer & Angeli, 2016). In a similar vein, Fredriksen (2016, 692) argues that efforts to prevent hybridisation of domestic cats and Scottish wild cats reflect a desire to preserve ‘static, set forms or essential categories’, at the expense of promoting change and ‘inventive life’.

Important questions to be asked are therefore:

1. Are anti‐hybrid arguments based on essentialist or purist thinking?
2. Does essentialism vary by conservation context?

​

### 2.3 Pragmatism

Systematics and efforts to preserve nature are both political practices. The extent to which conservationists care about admixture between taxa is therefore variable. For example, despite the finding that it was technically a ‘feral hybrid’, Indonesian primatologists described a macaque as endemic to the Togean islands because identifying a unique species was important for developing Indonesian conservation biology, attracting foreign donors and enlisting interest in the islands (Lowe, 2006). Similarly, protecting domestic cat/Scottish wildcat cross‐breeds may be strategically useful to prevent gamekeepers (who are legally entitled to cull feral cats) from killing wildcats and mostly wildcat hybrids (Langridge, 2020). Relatedly, in the Galàpagos, breeding between ‘immigrant’ and ‘resident’ finch species produced offspring with uniquely large beaks and bodies. These ‘big bird’ finches are ecologically successful and reproductively isolated from their parental types, and have been embraced by scientists (Lamichhaney et al., 2018).

Thus, conservationists may selectively weigh species indigeneity against taxonomic ambiguity, akin to ‘strategic essentialism’ employed by ethnic, national and other groups who simplify their shared characteristics when politically expedient (Eide, 2010). Whether we ‘care’ or not about hybridity is therefore shaped by socio‐economic and political circumstances. For example, conservation action plans tend to be directed towards the lowest levels of classification (i.e. subspecies and even populations; e.g. see IUCN/SSC Primate Specialist Group, 2020). This trend is perpetuated by the presence of multiple regionally dispersed initiatives that compete for attention and funding, stressing the uniqueness of their special charges (Palmer, 2020), which in turn fuels concerns about hybridisation. However, critics may argue that the uniqueness of small, isolated populations is overinflated, simply reflecting genetic drift rather than specific adaptations (Quilodrán et al., 2020), implying that action plans should be directed towards higher‐level taxonomic categories.

Another key factor shaping views is the increasingly pervasive effect of humans on the non‐human world, which makes it more difficult to preserve ‘untouched’ nature and to advocate a view of nature and society as separate. As populations decline due to rapid habitat modification, it may become impossible to maintain enough genetic diversity within species without passive (natural) or facilitated (human‐aided) intermixing of types. For example, wildfires in Australia have prompted considerations of assisted gene flow or genetic restoration to ensure the survival of the two to three subspecies of the emblematic koala (Seddon & Schultz, 2020). Thus, conservation in the Anthropocene could increasingly seek to employ ‘post‐natural’ and future‐oriented, ‘experimental’ approaches rather than preserve past environments (Collard et al., 2015; Fredriksen, 2016; Lorimer & Driessen, 2013; Rutherford, 2018).

However, critics worry that such approaches will lead to homogenisation and the loss of unique typologies, causing ‘speciation reversal’ (Kearns et al., 2018). Furthermore, post‐natural conservationists may be too eager to view ecosystems as resilient against anthropogenic threats, and to ignore the need to restore the environmental ‘ruins’ resulting from anthropogenic activities (Collard et al., 2015). Viewing humans and non‐humans as ‘entangled’ co‐creators of environments may also mask human dominance over other life (Giraud, 2019). In short, the argument that ‘nature is dead’ and what remains should be managed for the benefit of people, as suggested by some so‐called ‘new’ conservationists (Büscher & Fletcher, 2019), gives less incentive to restore and protect ecosystems. The erosion of the nature–culture divide could therefore help usher in further anthropogenic modification of the natural world, thus negatively affecting free‐living non‐humans, who are often clearly better off without anthropogenic interference (van Dooren, 2016). Thus, some ecomodernists argue that we should protect nature from further human activities, while others believe that technological solutions can and should be used to promote the coexistence of people with wildlife (e.g. using satellite telemetry to warn about approaching elephants; Venkataraman et al., 2005).

Important questions to be asked about pragmatism are therefore:

1. How do socio‐economic and political circumstances influence whether hybrids are embraced or ignored?
2. Does the erosion of ‘untouched nature’ render hybrids more important?

​

## 3 CASES: CHIMPANZEE AND ORANGUTAN SUBSPECIES HYBRIDS

To illustrate the ramifications of the outlined conservation‐related problems, we describe two case studies, based on our own research. Both involve the release of apes belonging to multiple subspecies into the same area, which led to hybridisations.

The first example, reflecting ethological and genetic research by J.N.M. (Msindai et al., 2021; Msindai & Sommer, 2021), refers to the chimpanzees *Pan* *troglodytes* of Rubondo island. In the early 1960s, Bernhard Grzimek, then director of the Frankfurt Zoological Garden in Germany, envisioned turning the island of Rubondo in the Tanzanian part of Lake Victoria in East Africa into a ‘sanctuary for threatened animals’ (Grzimek, 1970, 14). Bolstered by Grzimek's status as an honorary trustee of Tanganyika National Parks, various non‐endemic large mammals such as antelopes, elephants and colobus monkeys were transported between 1966 and 1973 to the 237 km^2^ island, which in 1977 became Rubondo Island National Park. The introductions also included 16 ex‐captive chimpanzees (7 males, 9 females) brought in from various European zoos, animal traders and circuses. The apes were set free without prior rehabilitation or training in foraging or nest‐building skills. Some mortality notwithstanding, the founder animals adapted to the wild and began to breed. Today, Rubondo is home to at least 35 chimpanzees. Population growth was aided by the absence of other apes and large terrestrial carnivores as well as effective protection from anthropogenic disturbance, with the natural forest cover intact (Huffman et al., 2008).

The currently recognised subspecies of *Pan troglodytes* are *P. t. verus* (West Africa), *P. t. troglodytes* (Central Africa), *P. t. schweinfurthii* (East Africa) and *P. t. ellioti* (Cameroon and Nigeria). Our own genetic analyses revealed that the Rubondo founder population included individuals from the subspecies *P. t. verus* and *P. t. troglodytes* (Msindai et al., 2021). Thus, the current population consists of hybrids of these two subspecies, while the nearest native range is that of *P. t. schweinfurthii*.

Our second case study refers to ape hybrids resulting from rehabilitation and reintroduction (R&R) of orangutans *Pongo pygmaeus* in Southeast Asia, based on multi‐sited ethnographic research by A.P. (Palmer, 2020). Since the 1960s, around 2,200 orangutans, former pets or rescued wild apes displaced by habitat destruction, were returned to forests of Borneo and Sumatra by R&R initiatives. Various taxonomic reclassifications have occurred since reintroduction first began (summary in Goossens et al., 2009). While Bornean and Sumatran orangutans were considered subspecies in the late 1960s, they were elevated to separate species in the early 2000s, with *P. pygmaeus* on Borneo and *P. abelii* on Sumatra. Soon after, three Bornean subspecies were described: *P*. *p. pygmaeus* in the northwest, *P. p. wurmbii* in centre and south, and *P. p. morio* in the northeast. However, the legitimacy of this taxonomy continues to be debated, given potentially as much genetic difference within as between subspecies (Arora et al., 2010). The description of a new species of orangutan south of Lake Toba on Sumatra, *P. tapanuliensis* (Nater et al., 2017), further complicates matters.

In any case, R&R projects have led to taxonomic admixture. Thus, while Tanjung Puting National Park is in the range of *P. p. wurmbii*, two female *P. p. pygmaeus* were inadvertently released into this part of Indonesian Borneo (Banes et al., 2016), an error that led to breeding between subspecies. Such admixture has likely happened elsewhere, including the possibility that Sumatran and Bornean orangutans were released on the wrong islands (Yeager, 1997).

In both our case studies, hybridisations were unintentional, aided by the fact that current subspecies classifications had not yet taken firm hold. While Rubondo island apes are unable to breed with native chimpanzees, the Bornean orangutans released into Tanjung Puting can mix with wild conspecifics. Both cases touch upon debates about the implications of hybridisations—including zoo management.

## 4 LESSONS: WHAT CHIMPANZEE AND ORANGUTAN RELEASES MEAN FOR HYBRIDS IN CONSERVATION

We now consider the implications of the Rubondo chimpanzees and Bornean orangutans for our key questions.

### 4.1 Breeding

#### 4.1.1 Do hybrids experience reduced reproductive success?

Hybrids may theoretically experience compromised survival and reproduction if their genetic make‐up differs with respect to the local adaptations of their parental lineages. Indeed, in both chimpanzees and orangutans, subspecies exhibit some typical *phenotypic* characteristics. In chimpanzees, face colours vary (bronze‐like in *P. t. schweinfurthii*, deep black in *P. t. troglodytes*, both black and white in *P. t. ellioti*, while the pink of *P. t. verus* newborns later turns dark), and *P.t. schweinfurthii* has the longest hairs. However, these variations might not be adaptations per se (Jenkins & Napier, 1976). Similarly, the larger size of western chimpanzees is thought to reflect ontogenetic scaling rather than fundamental differences in shape (Shea, 1984). However, at least eastern chimpanzees may possess unique genetic adaptations to viral pathogens (Schmidt et al., 2019). Furthermore, in theory intrinsic genetic incompatibilities may exist between subspecies, which may in turn lead to reduced reproductive output or fitness in hybrid offspring (Ely et al., 2005). Bornean orangutan subspecies likewise vary in coloration, with *P. p. morio* sometimes referred to as the black Bornean orangutan due to their dark pelage. Especially female *P. p. morio* also have larger jaws and smaller heads, potentially to cope with dietary stress (Taylor, 2009). Moreover, compared to Sumatran orangutans, Borneans in general show faster‐paced life histories, a trend most pronounced in *P. p. morio* (van Schaik, 2013).

As for *behavioural* traits, while chimpanzee populations differ in social customs and tool‐use pattern, (Whiten et al., 1999), these cultural variations are more expressed within subspecies than between (Kalan et al., 2020), perhaps reflecting that all shared a common ancestor until about 500 K years ago (Gonder et al., 2011). As for orangutans, Sumatrans tend to be more gregarious than Borneans. At a subspecies level, *P. p*. *morio* also exhibit the greatest reliance on non‐fruit fallback foods and smallest female home range sizes. Given that Bornean orangutan population substructures have emerged in just the last 80 K years (Nater et al., 2015), such differences are likely a product of behavioural plasticity rather than genetic variation (van Schaik, 2013).

There is no evidence of outbreeding depression on Rubondo; the chimpanzee population grew annually by at least 3.3%, one of the highest rates compared to native communities (Msindai et al., 2021). The ultimate outcome of the Tanjung Puting situation is inconclusive, as one of the two ‘misplaced’ *P. p. pygmaeus* females and her *P. p. pygmaeus* × *P. p. wurbmii* offspring had rather poor health and few offspring, while the other was highly successful (Banes et al., 2016). In zoos, the offspring of different chimpanzee subspecies have higher reproductive rates compared to non‐hybrids (Ely et al., 2005). Offspring of Sumatran and Bornean orangutans may experience reduced fertility and survival (Cocks, 2007), but there are no data on the outcomes of subspecies cross‐breeding. In short, there is less evidence that subspecies hybrids suffer from reduced survival and reproduction than evidence to the contrary (cf. also van Schaik, cited in Palmer, 2020,143; van Schaik, 2013).

#### 4.1.2 How are population‐level concerns and welfare of individual animals balanced in conservation breeding?

The hybrids resulting from releases on Rubondo and Bornean were not subject to specific management measures—if only because at the time current taxonomic classifications were not a matter of concern. However, captive ape hybrids are often targets of specific breeding policies, although there is no consistent approach.

For chimpanzee breeding programmes in the United States, subspecies identities are of no concern, neither for the roughly 400 zoo‐kept individuals [S. Ross, studbook keeper for the chimpanzee SSP (Species Survival Plan), pers. comm. to J.N.M.] nor in biomedical facilities (National Research Council Committee on Long‐Term Care of Chimpanzees, 1997). In contrast, since 2015, Japan's roughly 300 chimpanzees have been separated into *P. t. verus* and all the rest; the two groups are not permitted to breed (M. Huffman, pers. comm. to J.N.M.). Similarly, the European Association of Zoos and Aquaria (EAZA) is making efforts to genetically test the 728 chimpanzees in its accredited zoos. The 359 already tested individuals are separated into breeding pools for *P. t. verus* and *P. t. troglodytes*, while numbers of *P. t. schweinfurthii* and *P. t. ellioti* are considered too low to warrant coordinated action [F. Carlsen and T. de Jongh, chimpanzee EEP (SSP) coordinators, pers. comm. to J.N.M.). Already, since the early 2000s, a breeding moratorium was imposed for 102 identified hybrids, and this is now extended to all newly identified hybrids or not‐yet‐tested individuals. EAZA aims to reduce the number of hybrids, while growing the non‐mixed populations. However, a 2016 viability assessment established that under current management plans, *P. t. verus* would decline in the short‐ to mid‐term. Therefore, a few individuals that are not *P. t. verus* (with desirable behavioural skills or breeding experience) were selected to bolster the stock of the Western chimpanzees. For hybrids, the long‐term plan is nevertheless to reduce them to zero (F. Carlsen and T. de Jongh, pers. comm. to J.N.M.).

When Bornean and Sumatran orangutans were first identified as separate subspecies, captive‐bred hybrids were labelled ‘highly undesirable’ (van Bemmel, 1968, 14). It was feared that such ‘cocktail’ apes (Mallinson, 1978, 69) would divert resources for the production of ‘pure‐bred stock’ (Mallinson, 1978, 75) needed to create viable captive populations or 1 day repopulate the wild (Braverman, 2012). Therefore, in 1985 the orangutan SSP within the US‐based Association of Zoos and Aquariums (AZA) issued a moratorium on cross‐breeding, supplemented in 1994 by a recommendation to sterilise hybrids via surgery if housed in a situation where breeding could be possible (Orangutan SSP, 2015). At the same time, the SSP advised zoos to retain their hybrids, although some commentators proposed sending them to ‘retirement sanctuaries’ (Lindburg, 1991) or culling them (Lacy, 1995). At least some were sent to roadside attractions (Green, 1999). Today, while Bornean and Sumatran orangutans are separated, North American zoos do not manage Bornean orangutans at a subspecies level because of a lack of individuals or facilities (Orangutan SSP, 2015). In Europe, EAZA maintains that because zoos have been ‘mixing’ subspecies for a long time, there is little chance of unravelling admixtures (Bemment, 2018). Across Asia, no consistent management plan exists, not least because a 2018 study found that 14% of zoo‐kept orangutans were of completely unknown origin, while the assigned provenance of another 7% is questionable due to an unreliable test (Banes et al., 2018).

Thus, captive hybrid apes, like other ‘unloved’ subjects of conservation, often have their welfare compromised for the sake of the population. Being ‘consigned to genetic irrelevance’ (Chrulew, 2011, 150), they are prevented from breeding, which deprives them of a psychologically important experience (Asa, 2016; Braverman, 2012), and/or they are moved out of socially functional groups or housed alone. Thus, population‐level concerns trump the welfare of many individuals held in captivity.

### 4.2 Essentialism

We now consider whether anti‐hybrid conservation management reflects an essentialist or purist stance towards taxonomic units, and whether this varies depending on the context.

#### 4.2.1 Are anti‐hybrid arguments based on essentialist thinking?

Orangutan researcher Carel van Schaik views the prevention of mixing taxa in zoos as essentialist: it ‘sounds like this obsession with pure lines – it's almost a bit racist’ (cited in Palmer, 2020, 144–145). Van Schaik reasons that zoo orangutan hybrids probably do not suffer from compromised genetics and are unlikely to ever be released back to the forest. Zoo breeding programmes are commonly criticised because often captive populations are not genetically viable and there is no suitable release habitat, meaning that captive‐bred animals are hardly ever set free (Braverman, 2012). Some orangutans (Bullo, 2015) and chimpanzees (Grzimek, 1970; Hannah & McGrew, 1991) have been released from zoos, although arguably they did little or nothing for species preservation, given relatively large populations remaining in the wild (Beck, 2019; Palmer, 2020). Furthermore, if apes *are* reintroduced, priority should reasonably go to the at least 1,200 orangutans (Palmer, 2020; Sherman et al., 2020) and 1,100 chimpanzees (Trayford & Farmer, 2013) being kept in habitat‐country sanctuaries rather than zoos.

There is therefore little reason to think that maintaining genetically ‘pure’ populations in captivity is harmful to the apes, or necessary for conservation. Thus, there are reasons to view the exclusion or prevention of hybrids from zoo breeding programmes as essentialist or purist, a situation that has been likened to racism (Cribb et al., 2014, 228; see also Biermann & Mansfield, 2014; Fredriksen, 2016) and criticised by some scientists (Cribb et al., 2014, 228).

#### 4.2.2 Does essentialism vary by conservation context?

If it occurs ‘naturally’, mixing taxa is not commonly viewed as problematic (Allendorf et al., 2001; Stronen & Paquet, 2013). Among these unproblematic ‘natural’ hybridisations, chimpanzee subspecies *P. t. ellioti* have been known to mate with *P. t. troglodytes* in central Cameroon (Gonder et al., 2011; see also Bowden et al., 2012), and *P. t. troglodytes* with *P. t. schweinfurthii* near the Ubangi River in DR Congo (Hvilsom et al., 2013). On the other hand, mixing caused by humans (e.g. via reintroduction and translocation) is seen as interfering with the process of evolution (Palmer, 2020). For this reason, researchers such as van Schaik view the prevention of taxonomic admixture in reintroduction as desirable, if one can do it (in Palmer, 2020, 145–146).

However, even that argument is debatable, because humans have already significantly shaped the distribution and genetic structures of both chimpanzees and orangutans. In historic times, chimpanzees occurred across a wide belt of equatorial Africa (McBrearty & Jablonski, 2005), but were driven to extinction in Gambia, Benin, Burkina Faso, Togo and Zambia (Humle et al., 2016). Similarly, hunting and habitat modification after the late Pleistocene led to a decline in distribution and abundance of orangutans (Spehar et al., 2018). On Borneo alone, 150,000 individuals might have been lost between 1995 and 2015 (Voigt et al., 2018). Given this level of human interference in the non‐human world, the world is itself increasingly acknowledged to be a ‘hybrid’ co‐created by human and non‐human actors (Whatmore, 2002). This vision of nature and culture as intertwined underpins the field of ethnoprimatology, which maintains that non‐human primates rarely, if ever, live in a ‘natural’ setting free from human influence (Fuentes, 2012).

Anti‐hybrid policies in reintroduction could therefore be viewed as problematic for reinforcing a false dichotomy between nature and culture. Still, these policies are not essentialist per se, since they do not involve opposition to species change or the mixing of taxa. It would therefore be inaccurate to describe *all* anti‐hybrid policies in conservation as essentialist; prejudice against captive hybrid apes is perhaps essentialist, but efforts to avoid admixture in releases are not. Similarly, invasion biologists typically oppose non‐native species based on their impacts on economics and native species survival, rather than the xenophobia critics accuse them of (Simberloff, 2003; Switzer & Angeli, 2016). Thus, accusations of essentialism are perhaps too readily and uncritically made against conservationists opposed to taxonomic mixing and invasive species; in both cases, other important motivations may be at play, such as the desires to avoid meddling in nature or to lose native species. Both cases highlight that no single ‘conservation biopolitics’ is followed to manage the lives, reproduction and deaths of animals (Biermann & Anderson, 2017; Kiik, 2019). Hybrid management varies, depending on the conservation context.

### 4.3 Pragmatism

We now consider why conservationists might choose to ignore hybrid apes, and if the realities of the Anthropocene will result in hybrids being increasingly embraced or seen as unavoidable.

#### 4.3.1 How do socio‐economic and political circumstances influence whether hybrids are embraced or ignored?

As we saw in the case of zoos, chimpanzee hybrids are sometimes strategically employed to bolster the stock of small subspecies populations. Furthermore, as the isolated Rubondo island apes pose no ‘threat’ of mixing with other populations, they are not considered problematic. The same is true of other hybrids who may live on islands elsewhere in Africa. For example, in 1972–1979, captive chimpanzees were brought to the Mount Asserik area, Senegal and then transferred to three islands in the Gambia River in the Gambia (Hannah & McGrew, 1991). The apes were confiscated or donated and had previously spent time with animal traders, in research colonies, zoos or as household pets. The approximately 50 released chimpanzees grew to a population of about 120 today. While there are assumptions that the founders were from *P. t. verus*, there has been no genetic testing. Thus, these apes may well represent a mixture of two or more subspecies, similar to the Rubondo situation. Because they too are isolated on islands, their hybridity is of little concern to conservationists.

In Bornean orangutan R&R, hybrids are not isolated on islands. However, they nonetheless receive little attention, because separating subspecies poses numerous practical problems. Genotyping is expensive, and involves considerable paperwork as it is usually done in overseas laboratories (Banes et al., 2016). Moreover, although sanctuaries are already overwhelmed, finding release sites is a common challenge given the high cost and scarcity of suitable habitat (Palmer, 2020). One R&R initiative has multiple sites in different parts of Borneo, enabling them to transfer subspecies to the right parts of the island, presumably at considerable expense (BOSF, 2013). However, most groups are not in such a position. For example, Yayasan IAR Indonesia (YIARI) usually rescues *P. p. wurmbii* but occasionally finds *P. p. pygmaeus* orphans in need of R&R. Thus, a research and conservation advisor with YIARI explained that for a time they set about trying to secure two release sites, one for *P. p. wurmbii* and one for *P. p. pygmaeus*. Considerable efforts to find a second release site were unsuccessful, so YIARI now advocates to ‘have the subspecies scrapped’, that is, to challenge the taxonomy (cit. in Palmer, 2020:143).

The Indonesian government also acted pragmatically when accepting IUCN guidance from 2015, according to which Bornean orangutan subspecies can be released outside native areas (IUCN SSC Primate Specialist Group, 2015). This goes against general IUCN guidelines, which recommend that apes of unconfirmed subspecies identity should not be released, except under exceptional circumstances (Beck et al., 2007). However, a more recent Indonesian orangutan conservation action plan signalled the importance of maintaining genetic purity in protected areas, although it is unclear what this means in practice (KLHK, 2017).

#### 4.3.2 Does the erosion of ‘untouched nature’ render hybrids more important?

The IUCN classifies all orangutan species as Critically Endangered, and chimpanzees are classified as Endangered, with *P. t. verus* Critically Endangered—a situation that will likely deteriorate further (Hughes et al., 2011; Humle et al., 2016; Voigt et al., 2018). Under such circumstances, released populations may soon represent a substantial proportion of the remaining wild apes. Already reintroduced populations are identified as a conservation priority for Sumatran orangutans (Utami Atmoko et al., 2017), given their particularly low numbers in the wild (~14,000) and the large areas of forest into which releases were and are occurring. Perhaps the same will be true at some point of reintroduced populations on Borneo, even if they contain hybrids.

Furthermore, increasingly fragmented populations might require the introduction of genes from outside. Many local chimpanzee populations already harbour less than 100 individuals (Wittig & Boesch, 2019). Orangutan populations must contain perhaps 200 or even 500 apes to be viable (Kelle et al., 2013; Utami Atmoko et al., 2017). These constraints will likely lower the threshold to consider post‐natural approaches and to care less about taxonomic purity. In short, further population declines and habitat fragmentation could render hybrids more important for conservation in the future.

## 5 CONCLUSIONS: QUESTIONS ABOUT HYBRIDS IN CONSERVATION

Hybrids are unsettling, captivating, confusing—and also important. Mixed taxa force us to consider: (a) how to balance conflicting interests of individual animals, populations and ecosystems; (b) the basis on which boundaries between species and other taxonomic categories are drawn; (c) the underlying values of conservation, that is, what it aims to conserve; (d) the inescapability of the socio‐political contexts of taxonomy and conservation; and (e) if, how and why conservation should change in the Anthropocene. Therefore, rather than present recommendations, we conclude with a set of questions conservationists should ask themselves when making decisions about taxonomic hybrids.

### 5.1 Questions about breeding

#### 5.1.1 How similar is the behavioural repertoire of the animals in question?

Some species are further sub‐divided into different subspecies based on their present‐day geographic locality or selected genetic differences. However, there is sometimes little to no behavioural variation between such lower taxonomic ranks (e.g. chimpanzees), whereas subspecies of other taxa may differ more dramatically. Understanding differences between the animals involved would therefore help conservationists hypothesise whether hybridisation is likely to negatively affect survival and reproduction.

#### 5.1.2 How does breeding management affect welfare, and how should animal welfare be balanced with population‐level considerations?

Practices such as contraception, sterilisation and culling (which are commonly used for managing captive apes with undesirable genetics) impact upon the welfare of individuals. We therefore need to ask not only what these negative effects are, but also how much we should care about them, and whether it is worth compromising conservation to avoid these detrimental effects. The question of how to trade‐off individual animal welfare with population‐level concerns is a longstanding and difficult problem, with answers depending not just on facts but on values (Hargrove, 1992; Vucetich & Nelson, 2007). Answers to these questions may vary by species, with the welfare of individual hybrid apes more likely to attract concern than less charismatic and ‘unloved’ taxa, although opinions differ on whether such species favouritism is justified (Palmer, 2020).

#### 5.1.3 On what grounds are taxonomic boundaries drawn?

There are no uniform criteria of distinguishing species and other animal taxa. Furthermore, there is a political incentive to split rather than lump. It is therefore important to consider not only whether an animal is a hybrid, but also on what grounds the taxonomic classification has been made in the first place, and what this means for the likelihood that hybrids will experience a reduction in survival or breeding success. In other words, conservationists should seek to understand and evaluate the political contexts in which taxonomic classifications are made.

### 5.2 Questions about essentialism

#### 5.2.1 Given the context, are anti‐hybrid sentiments and practices based on essentialist, purist, even ‘racist’ thinking?

There is no single ‘conservation biopolitics’, and no single approach to managing hybridity across all conservation settings. While sentiment may sometimes be based on purist thinking (e.g. for apes in zoos), this is not true across all projects, such as reintroductions. This question therefore needs to be approached on a case‐by‐case basis. It is important for critics of essentialist thinking to bear in mind that other motivations may be at play when conservationists take issue with hybrids (e.g. reflecting a desire not to interfere with evolution), and in turn for conservationists to consider whether under the circumstances they could fairly be accused of essentialism.

#### 5.2.2 How common is ‘natural’ hybridisation?

Anti‐hybrid policies may rely on an ethic of not interfering with the natural world, rather than essentialism per se; thus, ‘natural’ admixture is often accepted while ‘artificial’ is opposed. However, it is not uncommon for animals of different taxa to cross‐breed in nature, thereby blurring the boundary between environmentally and human‐driven hybridisation. We therefore need to contemplate the causes and frequencies of cross‐breeding of the taxa in question. While we should avoid the naturalistic fallacy (i.e. because closely related taxa amalgamate in the wild, it is good and ought to be encouraged), documenting the extent of such ‘ordinary’ fusions would clarify whether in practice there is any ecological distinction between different hybridisation events.

#### 5.2.3 To what extent have humans already shaped the distribution, abundance and genetic composition of existing populations?

If historical anthropogenic influence shaped a taxon's genetic structure, this could be further grounds for questioning the natural/artificial distinction. In other words, in deciding how to preserve a perceived original state, conservationists should first reflect on the extent to which current taxonomic categories are themselves ‘natural’ (i.e. free from human interference). If population structures have already been significantly shaped by anthropogenic factors, then the idea of maintaining this genetic structure to preserve independent ‘nature’ makes little sense.

### 5.3 Questions about pragmatism

#### 5.3.1 How do socio‐economic and political contexts influence hybrid management?

The reality on the ground contributed to the little concern about subspecies mixing in the Rubondo chimpanzees and Bornean orangutans. In some contexts, it will not be practically feasible to worry about hybrids, and it may be politically desirable to embrace or ignore them. These contexts may shift over time, and should be considered when deciding what (if anything) should be done, including an assessment not only of the current context but also expected future changes.

#### 5.3.2 How will contextual elements change as the Anthropocene progresses?

Hybrids may become more valuable as the numbers and genetic diversity of free‐living apes and other non‐humans dwindle. We therefore need to look not only at the current situation, but also at what populations and conservation might look like in coming decades. Post‐natural conservationists therefore make a good point in arguing that it may be necessary in future to embrace compound organisms and novel ecosystems.

#### 5.3.3 To what extent is post‐natural conservation desirable?

It is increasingly recognised that a division between humans and nature is socially constructed and that future‐oriented, experimental approaches will be required in the Anthropocene. However, this could also be viewed as defeatist, offering little incentive to restore and protect ecosystems and failing to acknowledge that non‐humans are very often better off if they are separate from (rather than entangled with) people. It could also lead to homogenisation and the loss of unique typologies (i.e. everything could become a blend). Because post‐naturalism is an increasingly dominant mode of thought (Collard et al., 2015), we all have to think about how far down this track we are willing to go, and as such how much hybridity should be embraced.

There are no easy answers to any of these questions, and it will certainly be impossible to design a single, coherent conservation strategy for managing animals perceived to be composites. Only one thing is certain: there will be ongoing debate about hybrids.

## CONFLICT OF INTEREST

The authors declare no conflict of interest.

## AUTHORS' CONTRIBUTIONS

A.P. led the writing, framing and integration, and conducted the original research on orangutan conservation featured in the article; J.N.M. contributed substantial amounts of text, helped design the arguments and theoretical framing and undertook the original research on chimpanzees featured in the article; V.S. contributed text and constructively edited the manuscript, and supervised both research projects.

## Supporting information

Supplementary Material

## Data Availability

Due to the sensitive nature of A.P.’s ethnographic research, data will not be made publicly available.
