# Supplementary material for: Hybrid apes in the Anthropocene: Burden or asset for conservation?
Source: People Nat (Hoboken). 2021 May 12;3(3):573–86. doi: 10.1002/pan3.10214 (PMC8581989; doi:10.1002/pan3.10214)
Supplement: Supplementary file 1 — Supplementary Material [file PAN3-3-573-s001.pdf]

PEOPLE AND NATURE

Should conservationists worry about hybrid animals?

Alexandra Palmer, Volker Sommer, Josephine Nadezda Msindai

Conservationists often view hybridisation as problematic, at least if it was caused by humans. They therefore often aim to prevent breeding between, for example, domesticated and wild forms (e.g. wolves and dogs) and closely related animals that encounter each other in human-modified landscapes (e.g. coyotes and wolves). But critics suggest that we should be more tolerant and learn to love the 'monsters' we create, including hybrid animals.

In this paper, we glean insights into this debate using examples from our own research on chimpanzee and orangutan subspecies hybrids, in zoos and reintroduction projects. Hybrid apes, we suggest, are especially interesting and provocative because they're simultaneously unloved for their 'wrong' genetics, and loved due to the compelling charisma of our close kin.

We explore three key problems posed by hybrids. First, reproduction: hybrids are often assumed to experience poor survival and reproductive success, and they may suffer reduced welfare from captive breeding policies (e.g. sterilisation). Second, essentialism: anti-hybrid sentiment is often blamed on purist, even quasi-racist, thinking, yet the validity of this accusation may vary between contexts. Finally, pragmatism: hybrids might be strategically embraced or ignored in certain contexts, including when populations are sufficiently low or fragmented that hybrids become valuable for conservation.

Our ape examples illustrate that answering these complex questions demands a case-by-case analysis. For example, we suggest that accusations of essentialism make some sense in relation to zoo breeding, but fail to accurately characterise why conservationists try to avoid hybridisation in reintroduction. Furthermore, whether hybrids suffer reduced survival and reproductive success depends on a wide range of factors, including the political context in which taxonomic classifications were made (as we suggest, there's a political incentive to 'split' rather than 'lump' taxa). Answers to these questions will also depend on conservationists' values, for example whether they view as desirable or defeatist 'post-natural' approaches to conservation, which hold that conservation should be more future-oriented, experimental, and less concerned with preserving intact 'nature' (since this doesn't exist).

By setting out important these outstanding questions, and deriving lessons from apes, we demonstrate both the complexity and importance of the hybrid problem for conservation in the Anthropocene. One thing is certain: the issue isn't going away any time soon, and will only become more pressing in future.

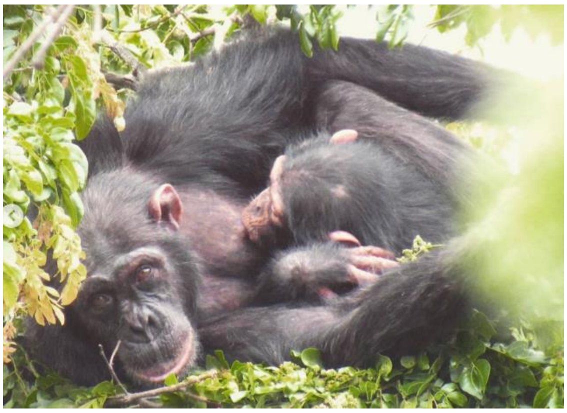

They say the eyes are the window to the soul, but can we tell these are hybrid chimpanzees? Photo of a Rubondo Island female with her infant in a nest, October 2019, Paul Kivuyo.
